# Why complicate an important task? An orderly display of the limb leads in the 12-lead electrocardiogram and its implications for recognition of acute coronary syndrome

**DOI:** 10.1186/s12872-018-0979-x

**Published:** 2019-01-10

**Authors:** T. Lindow, Y. Birnbaum, K. Nikus, A. Maan, U. Ekelund, O. Pahlm

**Affiliations:** 1 Department of Clinical Physiology, Växjö Central Hospital, Växjö, Sweden; 2 Department of Research and Development, Region Kronoberg, Sweden; 3 Clinical Sciences, Clinical Physiology, Lund University, Växjö, Sweden; 4 The Section of Cardiology, Baylor College of Medicine, and the Texas Heart Institute, Baylor St Luke Medical Center, Houston, TX USA; 5 Heart Center, Tampere University Hospital, Tampere, Finland; 6 Faculty of Medicine and Life Sciences, University of Tampere, Tampere, Finland; 7 Department of Cardiology, Leiden University Medical Center, Leiden, The Netherlands; 8 Clinical Sciences, Emergency Medicine, Skane University Hospital, Lund, Sweden; 9 Clinical Physiology and Nuclear Medicine, Skane University Hospital, Lund, Sweden

## Abstract

**Background:**

In the standard ECG display, limb leads are presented in a non-anatomical sequence: I, II, III, aVR, aVL, aVF. The Cabrera system is a display format which instead presents the limb leads in a cranial/left-to-caudal/right sequence, i.e. in an anatomically sequential order. Lead aVR is replaced in the Cabrera display by its inverted version, −aVR, which is presented in its logical place between lead I and lead II.

**Main text:**

In this debate article possible implications of using the Cabrera display, instead of the standard, non-contiguous lead display, are presented, focusing on its use in patients with possible acute coronary syndrome. The importance of appreciating reciprocal limb-lead ECG changes and the diagnostic and prognostic value of including aVR or lead −aVR in ECG interpretation in acute coronary syndrome is covered. Illustrative cases and ECGs are presented with both the standard and contiguous limb lead display for each ECG.

A contiguous lead display is useful when diagnosing acute coronary syndrome in at least 3 ways: 1) when contiguous leads are present adjacent to each other, identification of ST elevation in *two contiguous* leads is simple; 2) a contiguous lead display facilitates understanding of lead relationships as well as reciprocal changes; 3) it makes the common neglect of lead aVR unlikely.

**Conlusions:**

It is logical to display the limb leads in their sequential anatomical order and it may have advantages both in diagnostics and ECG learning.

## Background

ECG changes indicative of acute coronary occlusion or sub-occlusion serve as an important “watershed” in the early decision process in the management of patients with chest pain. ECG interpretation skills are therefore essential for any physician who encounters patients with suspected acute coronary syndrome. However, both accuracy and self-perceived confidence in ECG interpretation by internal-medicine and emergency-medicine residents have been reported to be low [1, 2].

**Fig. 1:**
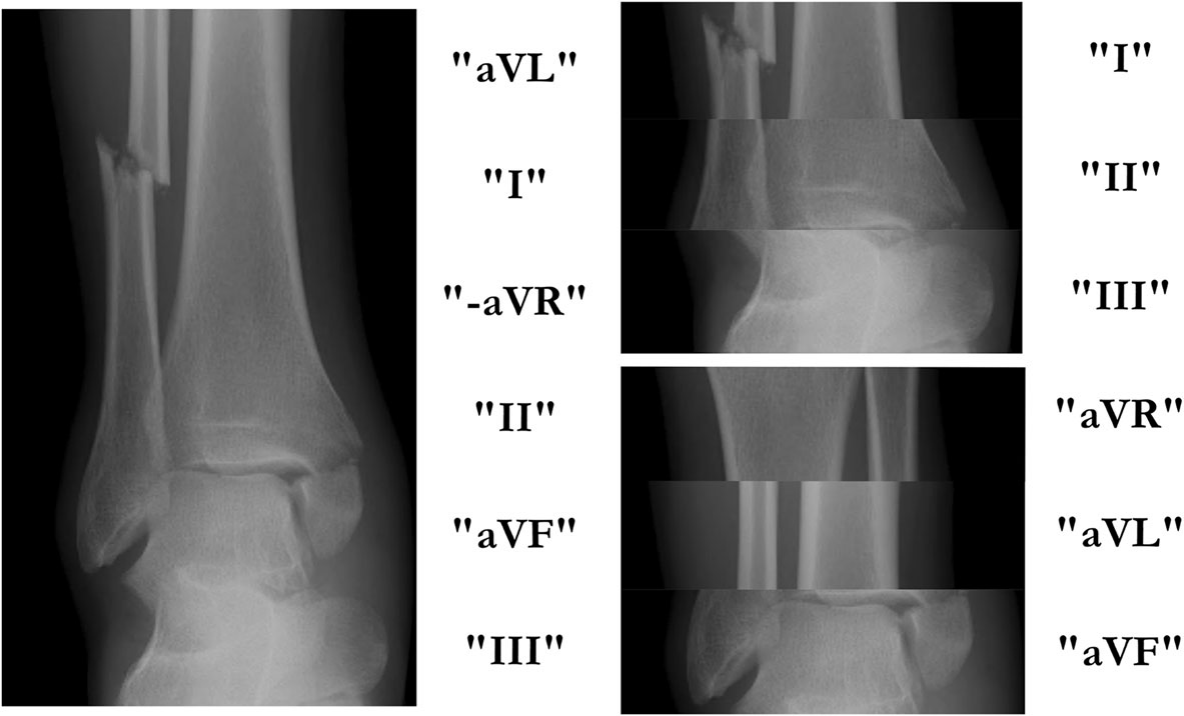
A figurative description of the illogical presentation of limb leads in the ECG standard format by the use of an X-ray image of a bimalleolar ankle fracture. The radiological image is divided into six parts with lead annotations to the right of each image in order to illustrate both the orderly and non-orderly lead sequences. To the left, the image is presented with the skeletal parts and the lead annotations in their orderly anatomical sequence. The right image is presented in scrambled order representing the standard, non-orderly presentation of the frontal plane sequence of the 12-lead ECG

In the standard 12-lead ECG, the precordial ECG leads are presented in an anatomical sequence, whereas the limb leads are most often presented as two groups of leads in a non-anatomical (“non-contiguous”) order: I, II, III and aVR, aVL, aVF (Fig. 1). This order of limb-lead presentation has historical roots. Leads I, II and III were the first ECG leads introduced by Einthoven in the beginning of twentieth century, and the augmented limb leads (aVR, aVL, aVF) were introduced later (by Goldberger in the 1940’s).

ECG waveforms in aVR are distinctly different from those in the other 5 leads, since it has its positive pole in the upper right quadrant of the thorax. Currents directed towards that positive pole will be represented as positive amplitudes in aVR, but as negative amplitudes in all other limb leads and vice versa. Thus, all reference values for aVR are completely different from those in the remaining leads. Q waves will become R waves, and ST elevation will become ST depression, which makes both teaching/learning and interpretation unnecessarily difficult.

**Fig. 2:**
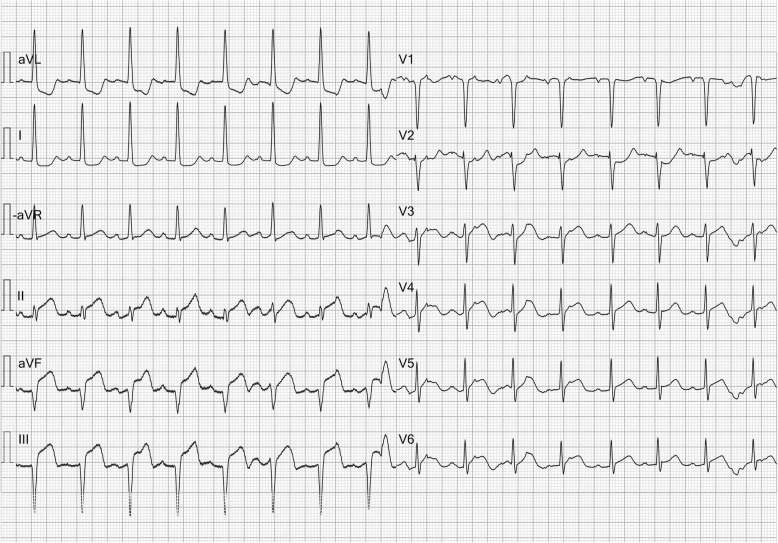
ECG presentation using the Cabrera sequence for limb leads (to the left), i.e. the limb leads are presented in an anatomically, contiguous sequence. The Cabrera sequence only affects the presentation of limb leads (aVL, I, -aVR, II, aVF, III). As in the standard display, the precordial leads are presented in contiguous order

Since the amplitude in aVF at any point in time equals the average amplitude of leads II and III it would be logical to display it between these 2 leads. In that way, it is easier to understand the waveform progression between leads. Likewise, the amplitude at any time point in the inverse of aVR, denoted −aVR, equals the average amplitude of leads I and II. Lead aVL equals the average amplitude of lead I and the inverse of lead III (−III). Hence, −aVR should logically be displayed between leads I and II, and aVL above lead I. Such a display is used in Sweden, the so-called Cabrera system, in which the limb leads are shown in a contiguous sequence at 30-degree intervals (Fig. 2) [3]. A contiguous lead display would be useful when diagnosing acute coronary syndrome in at least 3 ways: 1) when contiguous leads are presented adjacent to each other, identification of ST elevation in 2 contiguous leads is simple; 2) a contiguous lead display facilitates understanding of lead relationships; 3) it makes the common neglect of lead aVR unlikely. These aspects are explained and illustrated by ECG examples presented both with the standard, non-contiguous lead display and the contiguous display, the Cabrera sequence.

## Main text

### The Cabrera display for diagnosing acute coronary syndrome

The ECG is an essential diagnostic tool, since it is available very early in the management of patients with suspected acute coronary syndrome, often considerably earlier than biomarkers and other diagnostic modalities. The electrocardiographic diagnosis of ST-elevation myocardial infarction (STEMI) is based on presence of significant ST elevation in at least 2 anatomically contiguous leads [4]. In the standard presentation anatomically contiguous leads are not presented adjacently, which makes it unnecessarily difficult for the inexperienced ECG reader to apply the STEMI criteria. Only 3 lead pairs in the frontal plane can easily be recognized; aVL/I, II/aVF and III/aVF. In the Cabrera format, 2 additional lead pairs are evident; I/−aVR and − aVR/II.

### Reciprocal changes

**Fig. 3:**
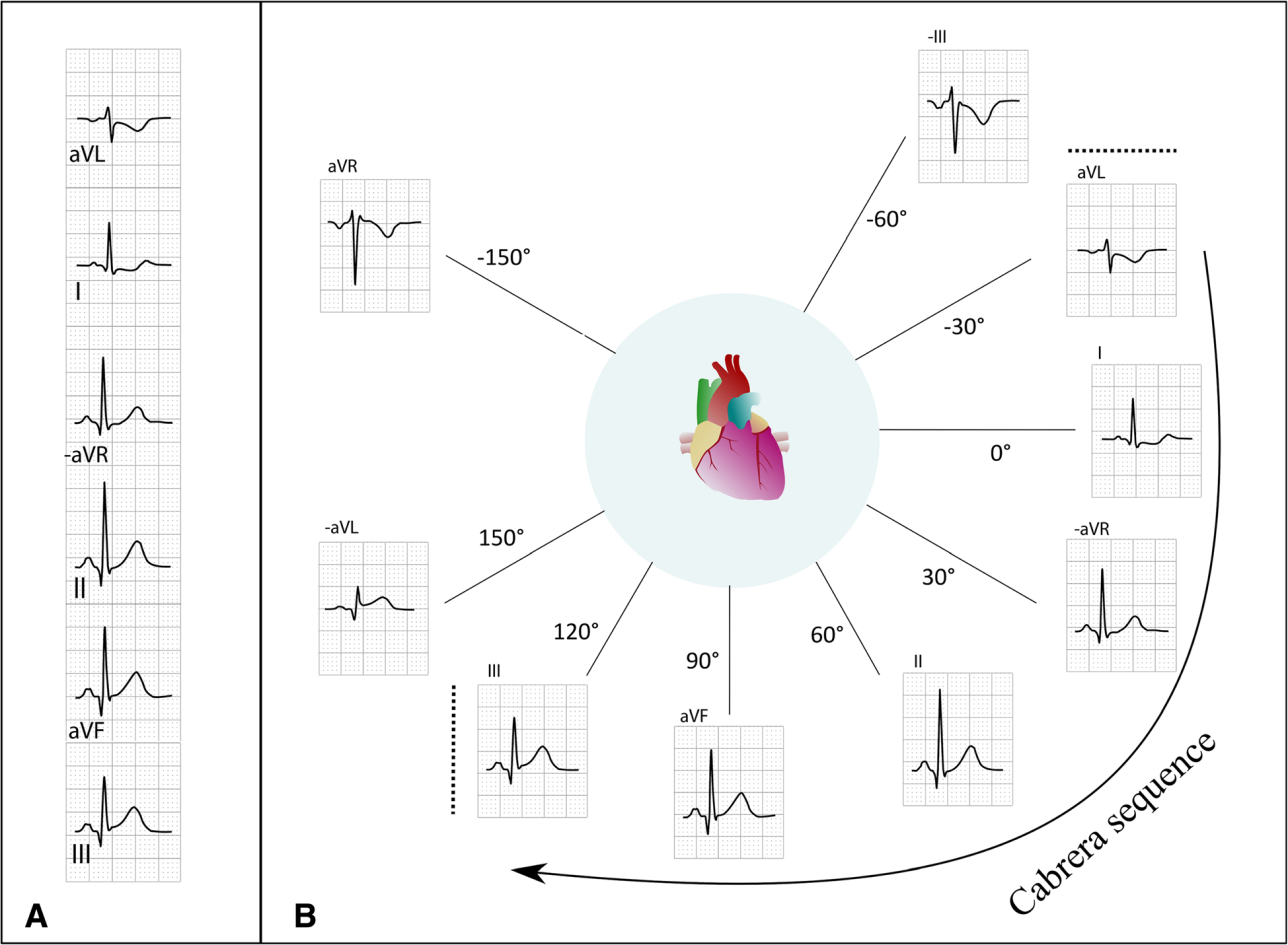
Panel A shows the Cabrera presentation of leads. The leads are presented in contiguous order. Compared to the standard format, aVR is replaced by –aVR. The relation between the limb leads according to the frontal axis is evident in Panel B. The Cabrera sequence is demarcated by the dashed lines above lead aVL and to the left of lead III. Lead aVR, which is included in standard display, is completely different from the other leads, with its positive pole in the upper right quadrant of the thorax. In the Cabrera display, the inverted version −aVR instead bridges the gap between lead I and II, resulting in a smooth waveform progression. This ECG, from a patient with RCA occlusion, shows significant ST elevation in only one lead, lead III, but reciprocal ST depression in aVL. Since −aVL is opposite to aVL and next to III, significant ST elevation can be considered to be present in two contiguous leads, III and − aVL

Identification of reciprocal changes in the ECG is important when interpreting ECGs in patients with suspected acute myocardial infarction. To identify these changes, it is necessary to understand how the limb leads are related. In an orderly display of the limb leads, it is easier to appreciate which leads are anatomically adjacent to each other. Reciprocal changes within the frontal plane, i.e. among limb leads, are also important. Lead aVL is almost reciprocal to lead III, which means that ST elevation in III may coincide with ST depression in aVL. The latter could be described as ST elevation in −aVL (the inversion of lead aVL). Lead III and − aVL, as well as aVL and − III are “neighboring leads” (Fig. 3b). This is likely well appreciated if the ECG is presented, and taught to medical students, with the leads displayed in contiguous order.

**Fig. 4:**
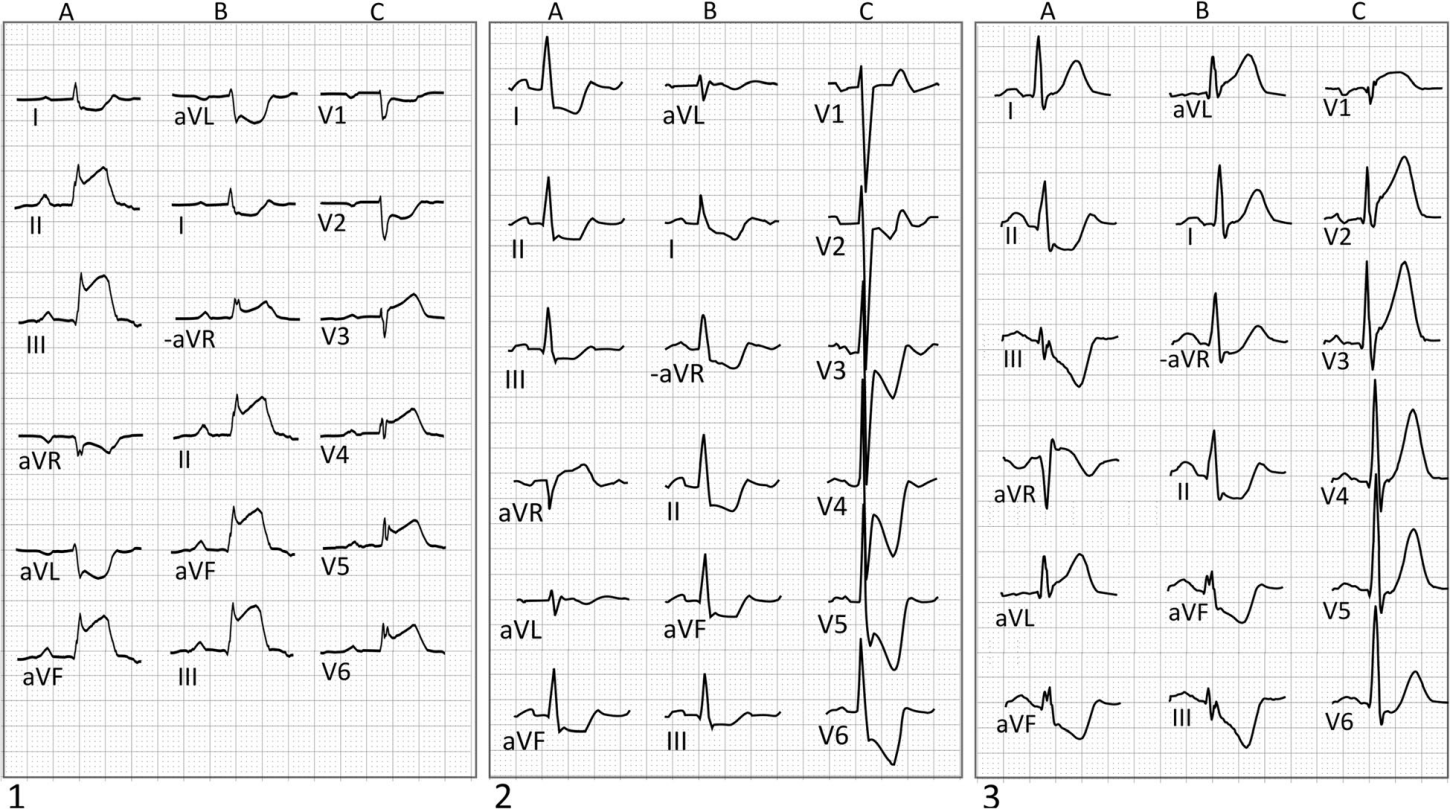
Three ECGs presented with both the standard presentation of limb leads (**a**) and the Cabrera sequence (**b**). Precordial leads are presented in columns **c**. 1. ECG from a patient with RCA occlusion. The myocardium at risk was large (37%) according to cardiac magnetic resonance imaging. There is ST elevation in II, III, aVF, V3 − V6 and ST depression in aVL, I and V1-V2. In the standard format, ECG shows significant ST elevation in two contiguous limb lead pairs; II/aVF and III/aVF. In the Cabrera format, ST depression in aVR is replaced by ST elevation in −aVR and ST elevation is thus evident in one additional lead pair, −aVR/II. The ST depression is larger in aVL than in I and the ST elevation is larger in III than in II. The inferior STEMI is easily recognized with the standard format, but in the Cabrera sequence, with −aVR presented adjacent to lead II, the extent of the infarction is more easily appreciated. 2. ECG from a patient with left main stenosis. Precordial leads show ST depression in V2 − V6 (**c**). In the standard format (**a**), ECG shows ST depression in I, II, III, aVF and ST elevation in aVR. In the Cabrera display (**b**), ST elevation in aVR is replaced by ST depression in −aVR with a smooth transition from the ST depression in its neighboring leads, in a way “de-mystifying” the ST elevation in aVR as a unique finding of severe coronary heart disease. 3. ECG from a patient with proximal LAD occlusion. ST elevation in anterior leads in combination with ST elevation in aVR in the standard presentation (**a**) is a sign of proximal LAD occlusion [16, 37]. In the Cabrera sequence (**b**), ST elevation in aVR is displayed as ST depression in −aVR, which makes it easy to appreciate the ST -segment progression. In the frontal plane, the ST vector is directed towards the left and ST elevation in aVL is present − a sign of LAD occlusion proximal to the first diagonal branch

ST changes in aVL carry important information in patients with inferior ST elevation [5, 6]. ST depression in aVL (i.e. ST elevation in −aVL) in inferior STEMI has been shown to indicate severe disease and poor left ventricular ejection fraction [7], and to increase detection rate in inferior STEMI [5, 8, 9]. ST depression > 0.1 mV in aVL is indicative of right ventricular (RV) infarction [10]. RV involvement in inferior STEMI is associated with increased risk of major complications, such as ventricular arrythmias, cardiogenic shock and death [11]. A recent study showed that differential diagnostic information may be added by assessing aVL in inferior ST elevation when differentiating between STEMI and pericarditis. In 154 STEMI patients, ST depression in aVL (at least 0.025 mV) was present in all patients, but in none of the patients with pericarditis [9]. Furthermore, ST depression in aVL and larger ST elevation in lead III than in lead II, indicates a right coronary artery (RCA) culprit (Fig. 4, left image) [12]. This can also be illustrated as a more rightward orientation of the ST segment deviation, with significant ST elevation in −aVL.

It is also important to include aVL in the interpretation of anterior ST elevation. When the left anterior descending coronary artery (LAD) is occluded proximal to the first diagonal branch, ST elevation in aVL and I is often present [13]. If LAD is long and “wraps” around the apex, ischemia of the inferior wall will also be present. If ischemia is present in both these areas, the “ischemic currents” of the lateral and inferior regions may oppose one another, reducing the number of leads with ST elevation [13]. This phenomenon is easily understood if one acknowledges that aVL is almost reciprocal to lead III (Fig. 3b).

### Neglect of aVR

Likely due to its different waveform morphology compared to the other limb leads, lead aVR is often ignored during ECG interpretation [14]. When 35 participants at an international scientific meeting on electrocardiography were asked to interpret 5 ECGs, the majority did not notice that aVR in all ECGs had been replaced by its inverted version [15]. Following this report, the importance of including aVR in ECG interpretation has been highlighted in several papers [14, 16–22]. ST elevation and positive T waves in aVR have been found to carry independent prognostic information [23, 24]. In patients with NSTEMI, ST elevation in aVR was associated with an increased risk of death, recurrent infarction and heart failure [24]. ST elevation in aVR in combination with ST depression in the precordial leads has been reported as a sign of three-vessel disease or left main coronary artery (LMCA) occlusion (Fig. 4, middle image) [14, 17, 25–30]. The widespread ST depression in combination with ST elevation in aVR seen in patients with left main stenosis reflects “circumferential subendocardial ischemia” [26]. This may also be present also in other conditions with an imbalance of oxygen supply and demand [26] and is therefore not specific for left main stenosis or three-vessel disease [17]. In patients with anterior STEMI, ST elevation in aVR > 0.05 mV can be a sign of proximal LAD occlusion (Fig. 4, right image) [31]. A proximal LAD occlusion site is associated with increased infarct size and increased short and long-term mortality, compared to mid- or distal occlusion sites, even in PCI-treated patients [32, 33]. In cases with ST elevation in aVR, a Cabrera presentation of the ECG would hence show ST depression in −aVR, with a smooth transition from the ST depression in its neighboring leads, in a way “de-mystifying” the ST elevation in aVR.

In inferior and lateral STEMI, presence of ST elevation in −aVR was associated with larger infarct size [22]. The left circumflex coronary artery (LCx) perfuses the lateral segments of the left ventricle, but overlap between RCA and LCx is common [34]. If an inferior STEMI is caused by an LCx occlusion or if the perfusion territory of an occluded RCA is large, ST depression in aVR will be present [35]. This is clearly visualized if leads are presented in contiguous order (Fig. 4, left image).

**Fig. 5:**
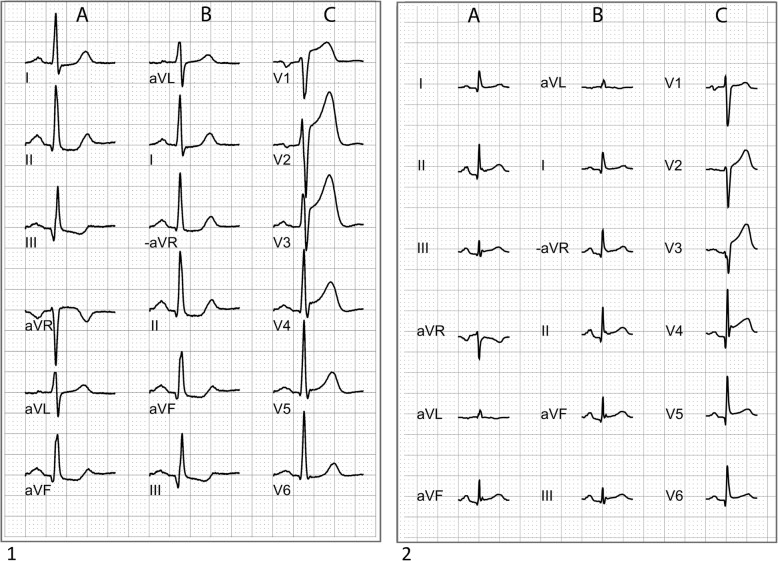
1. ECG from a patient with LAD occlusion. ECG shows ST elevation in V1 − V4 (**c**) and ST depression in II, III and aVF. In the standard format (**a**), slight ST elevation in aVR is present, displayed as ST depression in −aVR in the Cabrera sequence (**b**). 2. ECG from a patient with takotsubo cardiomyopathy. The ECG shows ST elevation in precordial leads (**c**) V3 − V6 and also ST elevation in II, III and aVF. In the standard format (**a**), ST depression in aVR is present, displayed as ST elevation in −aVR in the Cabrera sequence (**c**). ST depression in inferior leads and ST elevation in aVR (ST depression in −aVR) is more common in patients with LAD occlusion than in patients with takotsubo cardiomyopathy [37]. Kosuge et al. suggested that these differences were more easily visualized, and thus not as easily neglected, when −aVR was used and the leads were presented in contiguous order [37]

The use of aVR is considered to be important in the differential diagnosis between STEMI and takotsubo cardiomyopathy [36, 37]. Although ST elevation can be absent on admission ECGs in many cases of takotsubo cardiomyopathy, both the characteristics of the ST elevation, when present, and its time course are similar to those in acute myocardial infarction [36]. Kosuge et al. showed that in patients with ST elevation in the precordial leads, ST depression in aVR was more common in patients with takotsubo cardiomyopathy than in patients with LAD occlusion and suggested that this was more easily recognized by the use of the Cabrera sequence (Fig. 5) [37].

The focus of this debate paper is acute coronary syndrome. However, it deserves to be mentioned that including aVR in ECG interpretation has been described to be important for several other purposes; such as defining the origin of focal atrial tachycardia [38]; differentiating supraventricular [39] and ventricular wide QRS complex arrythmias [40]; and risk stratification of patients with Brugada syndrome [41]. The information in aVR is, of course, not lost by using its inverted version (−aVR), but instead probably less likely to be neglected when it is presented in its logical place between I and II.

As described above, when leads are presented in anatomical order, greater understanding of pathophysiological changes may be obtained. Hurst advised against interpreting ECGs by memorizing patterns and advocated that education in ECG interpretation should focus on understanding the spatial information in the ECG [42]. This is easier when leads are presented in the orderly Cabrera format. A study by Pahlm et al. showed that medical students identified the electrical axis faster and more correctly when using an ECG with the Cabrera display compared to the standard display [43], and the same would likely apply to the ST-segment axis in the frontal plane. Eslava et al. showed poor accuracy in ECG interpretation by internal medicine residents. ECGs representing acute myocardial infarction were the most correctly interpreted, but only 83% of those ECGs were correctly interpreted [1]. Besides the need of a structured ECG training curriculum [44], it is important that the ECG is displayed as logically and intuitively as possible, especially when ECG training for both students and clinical residents has to compete with other important clinical areas.

After the introduction of the Cabrera display in the 1950’s [45] several authors have recommended its use [46–51]. This display has been promoted in a position paper in the Journal of Electrocardiology [52], and was included as an appropriate alternative to the standard display in the recommendations for standardization of the electrocardiogram (2009) endorsed by both the American Heart Association and the American College of Cardiology [53]. It was successfully introduced in Sweden in the 1970’s but has gained less interest in other countries though it is used regionally in some countries, e.g. Finland, Japan, and Italy. Tradition and technical difficulties are the most likely reasons for the limited use of the Cabrera display. Nowadays, the technical difficulties are easily overcome since digital ECG software can present any display format as well as inverted leads.

Although the Cabrera sequence is an intuitive and logical presentation of limb leads, the change of display format would require an effort for many physicians in adapting to a new format, and comparisons of previous ECGs may become difficult unless the ECG software can switch easily between formats. Also, although lead −aVR has been included in clinical STEMI detection in Sweden for decades, scientific evidence on its use and its implications on decision making regarding reperfusion therapy is still lacking. Future studies on the value of the Cabrera sequence compared to the standard display format are needed and encouraged, especially regarding sensitivity and specificity for the diagnosis of acute coronary syndrome.

## Conclusions

It is logical to display the limb leads in their sequential anatomical order (Fig. 1), and it may have advantages both in diagnostics and ECG learning. Therefore, we encourage future studies on the value of the Cabrera sequence in ECG interpretation and education.

## Abbreviations

*LAD*: Left anterior descending coronary artery
*LCx*: Left circumflex coronary artery
*LMCA*: Left main coronary artery
*RCA*: Right coronary artery
*RV*: Right ventricular
*STEMI*: ST-elevation myocardial infarction
*NSTEMI*: Non ST-elevation myocardial infarction
